# Electrocardiographic pattern of apparently healthy African adolescent athletes in Nigeria

**DOI:** 10.1186/s12887-021-02557-8

**Published:** 2021-02-25

**Authors:** Ogochukwu J. Sokunbi, Christy A. N. Okoromah, Ekanem N. Ekure, Olajide A. Olawale, Wuraola S. Eke

**Affiliations:** 1 Department of Paediatrics, Faculty of Clinical Sciences, College of Medicine, University of Lagos / Lagos University Teaching Hospital, Lagos, Nigeria; 2 Department of Physiotherapy, Faculty of Clinical Sciences, College of Medicine, University of Lagos / Lagos University Teaching Hospital, Lagos, Nigeria; 3 Department of Nursing Services, Lagos University Teaching Hospital, Lagos, Nigeria

**Keywords:** Electrocardiography, Young athletes, Adolescents, African, Nigeria

## Abstract

**Background:**

Strategies to prevent sudden cardiac death (SCD) among young athletes have become topical worldwide and unrecognized cardiac pathology has been identified as a leading cause. Black ethnicity has been reported as an independent predictor of abnormal electrocardiography (ECG) findings among athletes and the frequency and significance of training-related ECG findings versus findings suggestive of an underlying pathology in the young African athletes is crucial.

**Methods:**

This cross sectional study aimed to determine the prevalence and distribution of ECG patterns in young athletes and controls. A total of 360 participants (180 athletes and 180 controls) were recruited from six secondary schools in Lagos, Nigeria between November 2014 and July 2015. Evaluation included interviewer-administered questionnaires for relevant history, physical examination and resting 12 - lead ECG for each participant.

**Results:**

Abnormal ECG patterns were found in 48.3% of athletes and 35.6% of controls. Training-related ECG findings occurred in 33.3% of athletes and 18.3% of controls. Athletes and controls had 7.7% prevalence of training un-related ECG patterns respectively. Left ventricular hypertrophy was the most common ECG finding among the athletes and male athletes had a higher prevalence of ECG abnormalities compared to females.

**Conclusion:**

Adolescent athletes in Nigeria have a high prevalence of training-related ECG patterns and athletes and non-athletes alike have similar proportions of ECG findings suggestive of underlying structural heart disease. Cardiovascular evaluation including ECG should be performed for young athletes prior to competition at any level and should also be considered as part of pre-school entry assessment for all children.

**Supplementary Information:**

The online version contains supplementary material available at 10.1186/s12887-021-02557-8.

## Background

The death of a young athlete during sporting activity is a most tragic and devastating event. Occult cardiovascular disease has been reported as the leading cause of sudden death in young athletes and about 56% of sudden deaths among competitive athletes aged 13 to 25 years are due to cardiovascular diseases [1]. Available data and reports also show that adolescent black athletes are most vulnerable to exercise-related sudden cardiac death (SCD) and tend to show a broader spectrum of ECG abnormalities compared to athletes of other ethnicities [2–4]. Strategies to prevent SCD in young athletes have been debated with regard to feasibility and efficacy of pre participation screening to detect underlying potentially life threatening cardiac diseases [5]; and the utility of the ECG has been questioned as trained individuals present a variety of changes, which in most instances are related to training and not of clinical significance [4]. In trained athletes, the 12-lead ECG shows a broad range of abnormal patterns, particularly increased QRS voltages, which are suggestive of left ventricular hypertrophy (LVH) and repolarization abnormalities [6, 7]. These alterations have been attributed to the physiological cardiac adaptations that occur as a consequence of systematic physical training [8] and the extent of these changes vary with the athlete’s gender, race, level-of-fitness, and type of sport [2, 9]. Distinguishing physiological ECG findings related to the athlete’s heart from changes suggestive of an underlying pathological cardiac disorder is critical to the identification of athletes at risk of SCD. Although there have been several recommendations and criteria to guide the evaluation of ECGs in athletes; further studies are needed to test the accuracy and utility of the present ECG criteria in relation to peculiar characteristics of athlete populations. It has previously been reported that sports in itself may not be the cause of enhanced mortality however, it has been shown to trigger cardiac arrest in those athletes who had cardiovascular conditions which predisposed to life-threatening ventricular arrhythmias during physical exercise [10]. Inclusion of a 12-lead ECG as part of pre-participation screening in addition to history and physical examination is being advocated globally [11, 12]. ECG screening among college athletes was noted to have a low false-positive rate and provided superior accuracy compared with a standardized history and physical examination to detect athletes with potentially dangerous cardiovascular conditions [13]. Although there is significant amount of information on ECG changes in professional athletes, there is a conspicuous lack of data on ECG changes in junior athletes in whom sudden death is more prevalent [14, 15]. Extrapolation of data derived from adult athletes for defining an abnormal ECG may be with an increased number of false-positive results, unnecessary investigations, and unwarranted anxiety. The limited information on the effect of black ethnicity on cardiovascular adaptation in adolescent athletes constitutes a major knowledge gap as both African origin and adolescence appear to confer a significant risk of SCD [16]. Although the updated guidelines for interpretation of athletes’ ECGs makes some allowance for African ethnicity, the criteria for further cardiac evaluation still rely on data from Caucasian athletes. The aim of this study was to characterize resting ECG patterns in adolescent athletes in Lagos, Nigeria who are involved in regular training for competitive sports.

## Methods

This study was carried out in six selected secondary schools in two local government areas in Lagos State, Nigeria. Lagos is a fast growing metropolis which generates about 25% of Nigeria’s total gross domestic product and is organized into 20 local government areas (LGAs) with an estimated population of 21 million people. The study was carried out over an 8 month period between November 2014 and July 2015. Schools were selected using convenience sampling. These schools fulfilled certain criteria which included having regular organized sporting activities for students, participation in inter collegiate sporting competitions and having qualified physical education tutors who could reliably identify athletes.

### Ethics statement

Ethical approval was obtained from the Health Research and Ethics Committee of the Lagos University Teaching Hospital, Idi Araba. Written informed consent was obtained from parents /guardians of participants and written assent was also obtained from all study participants prior to data collection according to the declaration of Helsinki for clinical research [17].

### Sample characterization (inclusion and exclusion criteria)

Thirty athletes and 30 controls were recruited from each school. Participants from each school were recruited by initial stratification of all the athletes in the school into six classes and subsequently systematic sampling within each stratum was done to get 30 subjects. Age, sex and body mass index matched controls were recruited from the different strata.

Athletes were defined as students involved in competitive intra-school or inter- school organized sport and who engaged in regular physical training in their various sporting disciplines at least three times a week and/or at least 3 h a week. These participants were matched for sex, age range and body mass index range with controls who had a relatively sedentary life style and were not involved in organized school sports. Exclusion criteria included symptoms suggestive of underlying cardiovascular disease, history of treatment or previous diagnosis of any chronic disease such as chronic renal failure, chronic liver or lung disease, sickle cell disease and/or regular consumption of alcohol or tobacco.

### Cardiac and sports participation survey questionnaire

All participants responded to a researcher administered questionnaire to obtain biodata, relevant cardiovascular history as well as information on the sporting discipline, duration and intensity of physical training and information was confirmed by the relevant coach / trainer in the sporting discipline for athletes and sectional heads for non-athletes. This questionnaire was developed for this study.

### Clinical assessment

Targeted general examination and cardiovascular assessment was carried out on all participants. Height and weight measurements were taken to the nearest 0.1 cm and 50 g respectively. Body mass index (BMI) was calculated as weight/height^2^ in units of kg/m^2^ and nutritional status of participants was classified using the WHO BMI-for-age charts.

### Resting 12-lead electrocardiography

A standard 12-lead ECG was performed during quiet respiration in the supine position for all participants using a Sonoscape SE 508 3 channel ECG recorder. Recording was done after each child had taken a 5 min rest on the couch and electrodes were placed carefully to ensure consistency of the precordial lead locations according to accepted recommendations [18]. ECG parameters were analyzed after considering the correct conversion factor for time and amplitude for the tracing using ECG calipers and a millimeter ruler where applicable [19]. The QT-interval was corrected for the heart rate (QTc) using the Bazzet’s formula [20] and LVH was identified using the Sokolow–Lyon criterion [21]. ECG patterns were evaluated according to the commonly adopted clinical criteria [9]. Specifically, the following criteria were considered as evidence of ECG abnormality: heart rate < 60 beats per minute (bradycardia) or > 100 beats per minute (tachycardia); prolonged PR interval defined as > 0.20 s was considered first-degree atrio-ventricular (AV) block; prolonged QTc interval was defined as QTc > 0.44 s in males and > 0.46 s in females; short QTc interval was considered as QTc ≤ 0.33 s; prolonged P-wave duration of > 0.10 s in standard leads I or II with negative portion of the P-wave ≥1 mm in depth and ≥ 0.04 s in duration in lead V_1_ was suggestive of left atrial enlargement; p wave voltage ≥0.25 mV was defined as right atrial enlargement; increased sum of S wave voltage in V_1_ and R wave voltage in V_6_ ≥ 35 mm in precordial leads was suggestive of LVH; RSR^1^ pattern in anterior precordial leads with QRS duration ≥0.12 s was consistent with complete right bundle branch block (RBBB); RSR^1^ pattern in anterior precordial leads with QRS duration < 0.12 s was defined as incomplete RBBB; QRS axis deviation ≤ − 30^0^ was suggestive of left anterior fascicular block (LAFB) and the left bundle branch block (LBBB) was defined as QRS duration ≥0.12 s; T-wave inversions and ST segment depression (≥2 mm) in ≥ two contiguous leads other than in leads V_1_, aVR and III and the early repolarization pattern evidenced by the upward ST-segment elevation ≥0.1 mV from baseline in ≥ two limb or precordial leads beginning from an elevated J point and continuing with an up sloping shape into the T-wave; Q wave was considered abnormal if it exceeded 0.04 s in duration and/or if the depth of the Q wave exceeded 25% of the height of the R wave and T wave height greater ≥11 mm was considered “tall”. ECG patterns were classified as normal (no abnormality), Group 1 (training-related) and Group 2 (training-unrelated) as previously described [22]. ECG quality assurance during the study included validation of waveform quality by two senior paediatric cardiologists experienced in ECG interpretation and performance of repeat ECGs when necessary.

### Statistical analysis

The primary outcome measure was identification of any abnormality on the ECG. Statistical analysis was performed using the SPSS statistical software version 17.0 (SPSS Inc., Chicago, IL, USA). Kolmogorov-Smirnoff test was used to test for normality. Differences between means were assessed with the Student’s t test and Mann-Whitney U test for normally and non-normally distributed variables respectively. Cohens’ *d* for T-test was used to measure effect size. The Chi square (χ2) test was used to compare the differences in proportions. Fischer’s exact test was used to compare proportions were appropriate. A 2-tailed *p* < 0.05 was considered statistically significant. The relationship between ECG abnormalities and BMI was assessed by stratifying the overall population into four groups (underweight, normal weight, overweight and obese) while the relationship of ECG abnormalities with age was assessed by categorizing the overall population into three age groups (i.e. 10–12, 13–15, 16–18 years).

## Results

### Socio-demographic characteristics of the study population

A total of 360 adolescents from six secondary schools were recruited for the study, 180 athletes and 180 non-athletes. The median age of participants was 15 years and in both study groups, male students made up 60% of the population.

### Clinical findings in the study population

Table 1 summarizes the clinical findings of the study participants. Patients with abnormal blood pressures on initial measurement had their blood pressure rechecked and all recruited participants were noted to have normal blood pressures on second reading. Two students who had repeatedly elevated blood pressure were counselled and referred for further care but were not included in the study. The mean BMI of athletes and controls was comparable, athletes had significantly lower mean pulse rates compared to controls (*p* < 0.001); tachycardia was twice more common among controls compared to athletes while bradycardia was more frequent in athletes compared to controls.

**Table 1:** Clinical findings in athletes and controls

Variables	Athletes	Controls	Test statistics	***P*** – value
**Mean age (years)**	14.46 ± 1.76	13.11 ± 1.75	t = 7.294	< 0 .001
**Mean BMI (kg/m**^**2**^**)**	20.13 ± 2.85	20.43 ± 4.19	t = − 0.782	0.435
**Mean oxygen saturation**^**a**^**(%)**	97.47 ± 1.32	97.77 ± 1.20	t = −2.29	0.220
**Mean pulse rate (bpm)**	81.93 ± 12.83	87.22 ± 13.91	t = −3.747	< 0.001
**Pulse rate categories**				
Bradycardia (< 60 bpm)	4 (2.2)	1 (0.6)		
Normal (60–100 bpm)	161 (89.4)	150 (83.3)	χ2 = 6.644	0.036
Tachycardia (> 100 bpm)	15 (8.3)	29 (16.1)		
**Blood Pressure (first measurement)**				
Low (<5th centile)^b^	1 (0.6)	3 (1.7)		
Normal (5th to 90th centile)^b^	163 (90.6)	170 (94.4)	χ2 = 4.669	0.097
Elevated (> 90th centile)^b^	16 (8.9)	7 (3.9)		
**Heart Murmur**				
Absent	176 (97.8)	180 (100.0)	χ2 = 4.045	0.044
Present (systolic ≤ grade 2/6)	4 (2.20)	0 (0.0)		
**BMI classification**				
Underweight	56 (31.1)	66 (36.7)		
Normal weight	112 (62.2)	93 (51.7)	χ2 = 6.767	0 .080
Overweight	11 (6.1)	15 (8.3)		
Obesity	1 (0.6)	6 (3.3)		

*bpm* Beats per minute, *BMI* Body mass index ^a^obtained by pulse oximetry ^b^derived from Blood pressure centile chart from the Fourth Report on the diagnosis, evaluation and treatment of high blood pressure in children and adolescents

### Sporting activities among athletes

**Fig. 1:**
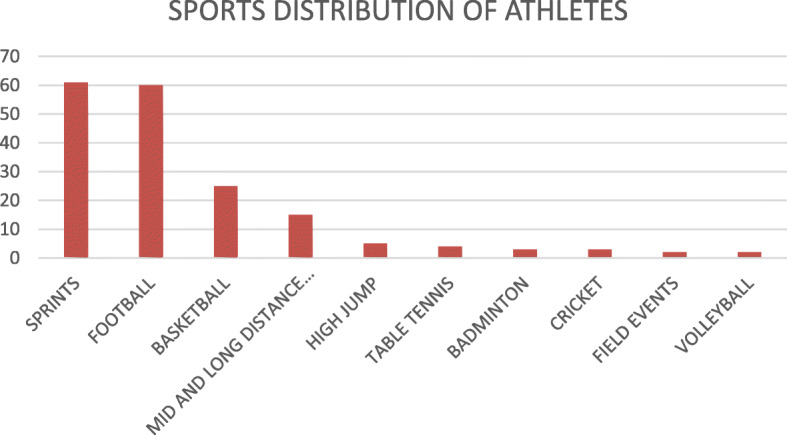
Bar chart showing the sporting disciplines represented by the athletes

The distribution of sporting disciplines and frequency of training activities is shown in Table 2. Athletes represented a variety of sport disciplines which were predominantly dynamic in nature with the modal sporting activities being football and sprints as shown in Fig. 1. Majority of the athletes recruited for this study (56.1%) participated in only one sporting discipline, while 55.0% competed in intra-school sport competitions alone compared to 45% of athletes who took part in both intra- and inter-school competitions.

**Table 2:** Sporting characteristics of athletes

Sporting Characteristic	Frequency	Percentage (%)
**Number of sporting disciplines participated in**		
1	101	56.1
2	45	25.0
3	21	11.7
4	10	5.6
5	3	1.7
**Cadre of competitive participation**		
Intra-school and inter-school	81	45.0
Intra-school alone	99	55.0
**No of training days per week**		
< 3	65	36.1
3–5	90	50.0
> 5	25	13.9
**Duration of regular training: no of hours per week**		
3	114	63.3
> 3, < 5	55	30.6
≥ 5	3	1.7
Unreported /unsure	8	4.4

### ECG patterns among athletes and controls

Among athletes, mean heart rate was significantly lower (*p* = 0.008), mean PR interval was longer (*p* = 0.003) and mean R wave voltage in V_6_ was higher (*p* = 0.013) when compared to controls. T wave amplitude in V_5_ was also significantly higher (*p* = 0.034) and R wave voltages in V_5_ were also higher among athletes compared to controls, although this was not statistically significant (*p* = 0.058). Table 3 shows the mean values of ECG variables of the study population.

**Table 3:** ECG patterns among athletes and controls

ECG variables	Athletes Mean ± SD	Controls Mean ± SD	Cohen’s ***d***	t test	***P*** value
**Heart rate (beats/min)**	80.16 ± 14.021	84.12 ± 14.100	0.282	−2.668	0.008
**PR Interval (msecs)**	158.17 ± 34.68	148.72 ± 25.33	0.311	2.951	0.003
**QTc interval (msecs)**	387.33 ± 27.69	387.60 ± 38.79	0.008	−0.075	0.940
**T wave amplitude V**_**5**_ **(mm)**	4.52 ± 2.21	4.09 ± 1.55	0.225	2.128	0.034
**T wave amplitude V**_**6**_ **(mm)**	3.44 ± 1.63	3.22 ± 1.39	0.145	1.374	0.170
**P wave amplitude (mm)**	1.60 ± 0.57	1.53 ± .490	0.132	1.197	0.232
**P wave duration (mm)**	80.39 ± 8.99	79.78 ± 7.32	0.074	0.707	0.480
**R wave in V**_**1**_ **(mm)**	4.40 ± 2.64	4.59 ± 2.38	0.075	−0.722	0.471
**S wave in V**_**1**_ **(mm)**	13.11 ± 6.09	13.30 ± 5.83	0.032	−0.301	0.764
**R wave in V**_**2**_ **(mm)**	9.19 ± 5.10	8.89 ± 3.94	0.065	0.613	0.541
**S wave in V**_**2**_ **(mm)**	16.83 ± 6.91	16.25 ± 6.35	0.087	0.834	0.405
**R wave in V**_**5**_ **(mm)**	19.16 ± 7.56	17.81 ± 5.70	0.201	1.906	0.058
**S wave in V**_**5**_ **(mm)**	2.46 ± 2.87	1.96 ± 2.38	0.189	1.791	0.074
**R wave in V**_**6**_ **(mm)**	14.77 ± 5.73	13.4167 ± 4.53	0.261	2.485	0.013
**S wave in V**_**6**_ **(mm)**	0.91 ± 1.16	0.78 ± 1.23	0.108	1.101	0.272
**QRS duration (msecs)**	81.28 ± 9.43	79.53 ± 8.38	0.196	1.867	0.063

*msecs* Milliseconds, *mm* Millimeters

### Prevalence of abnormal ECG patterns among athletes and controls

**Fig. 2:**
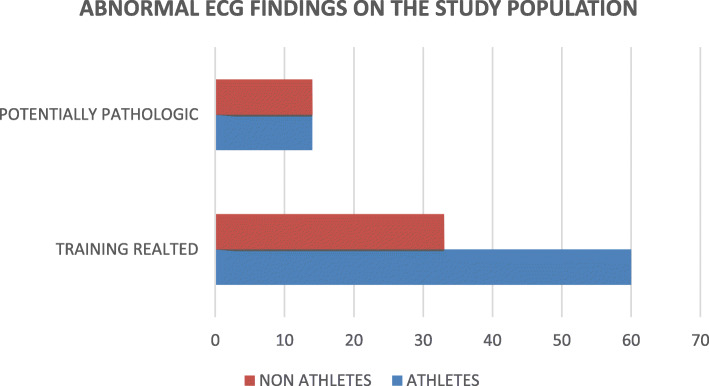
Bar chart showing proportions of abnormal ECG findings among athletes and controls

Abnormal ECG patterns were found in 48.3% of athletes and 35.6% of controls (χ2 = 6.034, *p* = 0.014) but training related ECG findings were seen in 33.3% of athletes and 18.3% of controls. Figure 2 shows the proportion and classification of abnormal ECG findings among athletes and controls.

In order of decreasing frequency, the most common ECG findings among athletes were LVH, IRBBB, first degree AV block and sinus bradycardia; while among controls sinus arrhythmia, sinus tachycardia, LVH and IRBBB were the most frequent ECG patterns.

Table 4 compares the prevalence of Group 1 ECG findings between athletes and controls. Presence of first degree AV block, LVH, sinus bradycardia and sinus arrhythmia was significantly different in both groups. Repolarisation changes were more common in athletes than non-athletes.

**Table 4:** Group 1 (training related) ECG findings in athletes and controls

ECG Abnormality	Athletes (***N*** = 180) n (%)	Controls (***N*** = 180) n (%)	Test statistic χ2	***P*** value
**First Degree AV Block**				
Present	9 (5.0)	1 (0.6)	6.583^a^	0.020
**LVH**				
Present	20 (11.1)	7 (3.9)	6.767	0.009
**Sinus Bradycardia**				
Present	9 (5.0)	1 (0.6)	6.583^a^	0.020
**Ectopic atrial rhythm**				
Present	0 (0.0)	2 (1.1)	2.011^a^	0.499
**Junctional Rhythm**				
Present	0 (0.0)	1 (0.6)	1.003^a^	0.500
**IRBBB**				
Present	11 (6.1)	6 (3.3)	1.543	0.214
**Sinus Arrhythmia**				
Present	5 (2.8)	14 (7.8)	4.501	0.034
**Early Repolarization changes**				
Present	6 (3.3)	1 (0.6)	3.642^a^	0.121
**Total**	60 (33.3)	33 (18.3)		

*IRBBB* Incomplete Right Bundle Branch Block, *LVH* Left ventricular hypertrophy

^a^Derived by Fisher’s exact test

The prevalence of potentially pathologic ECG findings in athletes and controls is depicted in Table 5.

**Table 5:** Group 2 (potentially pathologic) ECG findings in athletes and controls

ECG Abnormality	Athletes (***N*** = 180) n (%)	Controls (***N*** = 180) n (%)
**Left axis deviation**	1 (0.6)	0 (0.0)
**Prolonged QTc interval**	3 (1.7)	2 (1.1)
**Short QTc interval**	3 (1.7)	2 (1.1)
**Short PR interval**	4 (2.2)	0 (0.0)
**Right atrial enlargement**	1 (0.6)	1 (0.6)
**Left atrial enlargement**	1 (0.6)	1 (0.6)
**Tall T waves in V**_**5**_**, V**_**6**_	1 (0.6)	8 (4.4)
**Total**	14 (7.7)	14 (7.7)

In both athletes and controls, 7.7% of each group of participants had ECG findings which were considered training-unrelated and potentially pathologic (Group 2).

### Relationship between abnormal ECG findings and selected variables

The relationship between abnormal ECG findings and three variables: gender, age and BMI is shown in Table 6. Male athletes and controls had higher prevalence of abnormal ECG findings when compared to female athletes and controls respectively (*p* = 0.038 and *p* = 0.036 respectively). First degree AV block and LVH were significantly more common in male athletes compared to female athletes (*p* = 0.030 and *p* = 0.04 respectively). Although IRBBB was most common in the youngest athletes aged 10 to 12 years (χ2 = 8.14, *p* = 0.017), there was no significant difference between the presence of abnormal ECG findings and age groups in both athletic and non-athletic study populations. The proportions of athletes and controls who were overweight and underweight respectively were similar in both athletic and non-athletic populations and BMI was not significantly associated with the occurrence of abnormal ECG findings in athletes or controls respectively. (*p* = 0.438, *p* = 0.853 respectively).

**Table 6:** Relationship between abnormal ECG findings and variables

Variables	No of abnormal ECGs		Total
	Athletes (***N*** = 180)	Non-Athletes (***N*** = 180)	
**Gender**			
Male	59 (67.8)	45 (70.3)	104
Female	28 (32.2)	19 (29.7)	47
Total	87 (100.0)	64 (100.0)	151
Test Statistic	χ2 = 4.286, *p* = 0.038	χ2 = 4.401, *p* = 0.036	
**Age groups**			
10–12	16 (18.4)	30 (46.9)	46
13–15	39 (44.8)	29 (45.3)	68
16–18	32 (36.8)	5 (7.8)	37
Total	87 (100.0)	64 (100.0)	151
Test Statistic	χ2 = 2.379, *p* = 0.304	χ2 = 1.982, *p* = 0.371	
**BMI**			
Underweight	23 (26.4)	23 (35.9)	46
Normal	57 (65.5)	32 (50.0)	89
Overweight	6 (6.9)	6 (9.4)	12
Obesity	1 (1.2)	3 (4.7)	4
Total	87 (100.0)	64 (100.0)	151
Test Statistic	χ2 = 2.715, *p* = 0.438	χ2 = 0.957, *p* = 0.853	

*BMI* Body Mass Index

The highest frequencies of abnormal ECG findings were seen among football and basketball players. All cricket players had abnormal ECG patterns while none of the athletes who played badminton and table tennis were noted to have abnormal findings on ECG. However, the athletes who participated in cricket, badminton and table tennis were less than 10 in number.

**Fig. 3:**
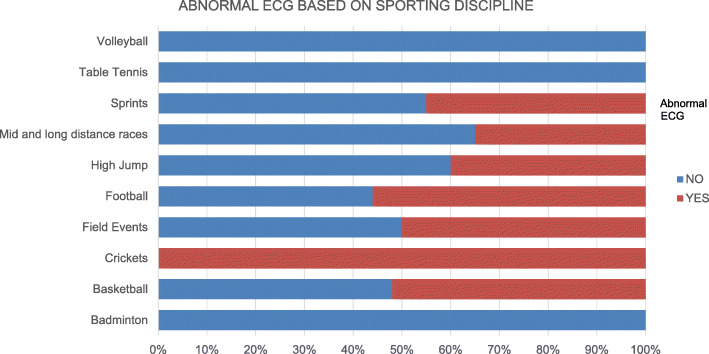
Bar graph showing frequency of abnormal ECG findings based on sporting disciplines

Figure 3 shows the frequency of abnormal ECG findings based on sporting disciplines.

The association between abnormal ECG patterns and sporting characteristics among the athletes is shown in Table 7. The number or type of sporting disciplines, cadre of participation and the duration of regular training per week was not shown to be associated with the presence of abnormal ECG findings.

**Table 7:** Frequency distribution of abnormal ECG findings according to sporting characteristics of athletes

Sporting Characteristics	Abnormal ECG		Total n(%)	Test Statistic
	No n(%)	Yes n(%)		
**Number of sporting disciplines**				
≤ 2	73 (49.5)	73 (50.5)	146	χ2 = 0.860
> 2	20 (51.1)	14 (48.9)	34	*p* = 0.354
Total	93 (51.7)	87 (48.3)	180	
**Cadre of competitive participation**				
Intra-school and inter-school	38 (46.9)	43 (53.1)	81	χ2 = 1.332
Intra-school alone	55 (55.6)	44 (44.4)	99	*p* = 0.248
Total	93 (51.7)	87 (48.3)	180	
**No of training days per week**				
≤ 3	64 (50.8)	52 (49.2)	116	χ2 = 1.606
> 3	29 (52.2)	35 (47.8)	64	*p* = 0.205
Total	93 (51.7)	87 (48.3)	180	
**Duration of regular training: no of hours per week**				
≤ 3	84 (53.5)	76 (46.5)	160	χ2 = 0.525
> 3	5 (47.3)	7 (52.7)	12	*p* = 0.469
Total	89 (51.7)	83 (48.3)	172	

## Discussion

### Main study findings

We report the pattern of ECG findings in apparently healthy adolescent athletes and controls of Nigerian descent. Abnormal ECG patterns were significantly more common among athletes compared to controls. Training-related and benign ECG findings were more common among athletes, while both athletes and controls had the same prevalence of training- unrelated and “potentially pathologic” ECG patterns Pellicia et al [9] and Sharma et al [23] reported similar frequencies of abnormal ECG findings among highly trained junior elite athletes as was reported in this study. Prevalence of ECG abnormalities in different studies may vary based on the criteria used for classification of the ECG as “normal” or “abnormal”; for example the inclusion of some benign ECG findings such as IRBBB, first degree AV block and sinus bradycardia as normal in the Italian study [9]; while these findings were considered as abnormal but benign and training-related in the current study. Utilization of different criteria in the same cohort of participants has previously been shown to reduce the proportion of abnormal ECGs [24]. Over the years, there have been several revisions of criteria utilized in interpretation of the athlete’s ECG, in a bid to reduce false positives and improve the specificity for ECG detection of potentially pathologic cardiac conditions [25].

### ECG findings and ethnicity

It has been advocated by several authors that studies are needed to examine racial differences in the performance and utilization of different assessment tools during pre- participation cardiovascular screening in athletes [26]. Ozo and colleague [27] reviewed the impact of ethnicity on cardiac adaptation in athletes of various origins and highlighted some of the ECG changes known to be more commonly found in black athletes such as higher prevalence of ventricular hypertrophy and repolarization changes.

The prevalence of potentially pathologic ECG findings from the present study did not vary significantly from the proportions reported among athletes of other races [3]. Although athletes of African origin have previously been reported to demonstrate a significantly greater rate of abnormal ECG changes compared to other ethnic groups, it has been argued that these disparities are more likely to be related to the type and implementation of pre-participation screening leading to the identification of athletes at risk, rather than reflecting a truly different ethnology [28]. Isolated criteria for left ventricular hypertrophy using QRS voltages was the most common ECG pattern documented in athletes in this study and about 4% of controls fulfilled this same criteria. Perhaps this finding is akin to both groups by virtue of their common African descent. The presence of similar ECG findings in the study and control groups of the index population, brings to light the possibility that some of these training related ECG findings may be present in some black adolescents regardless of their level of sports participation. While consideration has been given for presence of repolarization changes among black athletes in current guidelines, no special mention was made about criteria for LVH in these group of athletes [25]. The fact that isolated voltage criteria for LVH does not warrant further investigation may have contributed to the lack of its specific consideration. More studies on homogenous populations are needed to clarify the role of ethnicity in the prevalence of disorders known to cause SCD in young athletes.

### Level of sports participation

Athletes in this study spent an average of 3 h per week training in sporting activities on a regular basis. The index population were school children within a structured curriculum; thus more hours of regular training may not have been practicable. In an environment where there are little or no significant merits for sporting excellence in high schools, the time devoted to regular training is largely limited; unlike some parts of the world where a high premium is placed on sports participation as it has a potential to be highly rewarding. Participants engaged in training activities at least three times a week, giving a fairly regular frequency of sports participation; and although the number of hours spent on organized training among the index cohort is known, the intensity of sporting activities which occurred during these training periods could not be objectively quantified. While most athletes engaged in more hours of training during the sports season, the study focussed on the duration of regular training. Participation in competitive sports implied some level of intensity in performance of sporting activities and this was utilized in the index study as a measure to suggest premium on performance which is one of the key characteristics of athletes [25, 29]. Training related ECG changes are known to be more common in athletes who engage in at least 4–8 h of intensive exercise weekly; nevertheless prudent application of current guidelines among individuals with lower levels of exercise has been advocated.

### Training related ECG findings in the study population

The spectrum of abnormal ECG findings in the current study was similar to that previously documented among athletes and controls [30] using similar guidelines. In both studies, group 1 ECG abnormalities were more prevalent and in keeping with features of the athlete’s heart which have been extensively described in literature [31]. QTc anomalies were the predominant pathological ECG finding. About one-tenth of control participants were found to have training-related ECG patterns. Some group 1 ECG abnormalities are common findings in healthy children and this may explain the high prevalence even among non-athletic controls. It is also possible that the non-athletes in the present study might have had a fairly active nature thereby rendering it a mixture of recreational athletes and more sedentary individuals rather than a purely non-athletic group. This is suggested by the routine non-competitive out door physical activity which all students are mandated to participate in at least once a week in the secondary schools where the study was conducted.

### Potentially pathologic ECG findings in the study population

The present study reported similar prevalence of training-unrelated ECG findings among athletes and controls as in two previous South Asian [32] and Italian [33] studies respectively. It is worthy of note that cardiac pathology as suggested by the ECG may be detected in athletes and non-athletes alike [26]. Over the years, screening efforts have been geared towards athletes alone based on the premise that individuals engaged in competitive sports are at significantly higher risk for SCD compared to individuals of similar age who are not involved in such athletic activities and lifestyle [10, 11, 13]. However, catastrophic events have been documented to have a higher incidence in non-athletes than athletes [34]. It is thus necessary to bring to the fore the need to suspect, search for and identify potentially pathologic ECG patterns even in the non-athletic population and it appears only logical and ethical that some consideration ought to be given to include non-athletes in screening programs. More so, since mass examination initiatives have not been advocated for the much larger general population of children and adolescents who may not participate in organized sports, there is little data regarding the detection of cardiovascular disease from such cohorts and the true burden remains unknown [35].

### Specific ECG findings

#### Sinus arrhythmia

The prevalence of sinus arrhythmia in this study was thrice more common among controls than athletes. Sinus arrhythmia is a common finding in the paediatric age group so it was not unexpected that despite variation in ethnicity, there was a similar frequency of this ECG pattern among controls in this study as was documented in previous Caucasian studies [23, 36]. Among athletes, sinus arrhythmia has been reported with widely varying frequency, from 13 to 69% [37–40]. These variations likely reflect the individual athletes’ autonomic state and level-of-fitness as well as the definition of sinus arrhythmia used by the various authors. Higher prevalence than that found in the present study was reported among male veteran endurance athletes [40] and among professional football players [39] with presumable participation in higher intensity activities than the present study participants.

#### Sinus bradycardia

Sinus bradycardia was more frequent in athletes compared to controls with a prevalence of 5.0 and 0.6% respectively. The prevalence of sinus bradycardia among athletes in this study was much lower than 80% which was reported in a previous study in black athletes [22] and two cohorts of predominantly Caucasian athletes [23, 36]. Although the mean age of participants in the Caucasian studies [23, 36] were similar to that in the present study, the number of training hours put in by the Caucasian athletes (11 ± 4.5 h/week and 9.7 ± 3.3 h a week respectively) was much higher compared to athletes in the current study who spent an average of 3 h per week training in their various sport disciplines. This supports the hypothesis that bradycardia likely reflects the level of athletic conditioning [37]. In a similar population of children aged 8 to 18 years [33] the prevalence of sinus bradycardia (6.2%) was similar to the finding of the present study. A higher prevalence of sinus bradycardia in adults compared to children may not be unrelated to age given that heart rate is known to decrease with increasing age. Although bradycardia is recognized as an adaptive response in athletes, it is sometimes associated with acute cardiovascular events such as syncope, particularly when associated with AV block patterns. Thus, it may be necessary to further investigate bradycardia even among athletes especially in the presence of symptoms.

#### PR and QTc intervals

The average PR and QTc intervals among athletes in this study was similar to values reported previously [23]. Similar to our findings, Bjørnstad and colleauges [41] documented a significantly longer PR interval compared to controls supporting the general hypothesis that training-induced ECG changes may partly be due to alterations in autonomic tone and partly to structural changes in the myocardium in athletes [41].

#### Atrioventricular and bundle branch block

The proportion of athletes in this cohort with first degree atrioventricular (AV) block was comparable to a previous report by Papadakis among adolescent athletes [36]; IRBBB was seen in twice as many athletes than controls as previously documented [42]; and although IRBBB is common in the paediatric population, these findings suggest that IRBBB is likely partly consequent on athletic conditioning and not entirely due to age of the present study population.

#### Left ventricular hypertrophy

Eleven percent of athletes in this study had isolated QRS voltages which fulfilled the criteria for LVH; similar to findings by Langdeau et al. [43] in a study involving athletes and non-athlete groups. Much higher prevalence (45–49%) have however been reported even among adolescents [36, 42]. It has been previously suggested that LVH could be attributed to normal, long term adaptation to intense, repeated exercise [9], and it is arguable that more highly trained student-athlete populations, than the index cohort studied, would have a relatively higher rate of LVH. While several theories attempt to explain the occurrence of LVH in athletes, the absence of ST segment depression, T wave inversion, pathologic Q waves and left axis deviation as noted in the study group reduces the likelihood of pathologic hypertrophy and likely reflects a benign chamber enlargement. LVH was found in few non-athletes as well. QRS voltages are known to be affected by the properties of the chest wall with slimmer persons having higher QRS voltages and this may explain the presence of this ECG finding even among non-athletes [23]. Although both study groups had similar body surface areas, athletes may likely have had thicker chest walls which may cause less exaggeration of QRS voltages and thus, LVH in these athletes may be a true reflection of cardiac conditioning in response to exercise.

### Association between ECG findings and age, sex and BMI

In this study, male sex was significantly associated with abnormal ECG patterns, further supporting previous reports that ECG abnormalities are more common in male than in female populations [5, 30]. It is likely that male predominance in many sporting activities with usual consideration for the degree of exercise intensity female athletes are permitted to be exposed to, may explain this. Age did not affect the presence of abnormal ECG patterns in this study. The variations in the paediatric ECG are known to approach adult values in the pubertal/ adolescent period which is the age range of the participants in this study. Expectedly, IRBBB which is a normal finding in children [44] was more common in the youngest group of athletes in this cohort. Body mass index was not significantly associated with ECG findings in this study and although it may be expected that cardiac morphology would be affected by body mass, particularly in obese individuals; the relatively small proportion of overweight and obese participants in the present study may explain this lack of association.

Pre-participation screening of athletes using the ECG is often less straight forward than assumed. This study has highlighted the peculiarities of the athletes’ heart among an adolescent population of African descent with a comparatively lower level of exercise compared to professional athletes. Performance and interpretation of the athlete’s ECG requires skill and use of current recommendations to avoid false positives and false negatives with their attendant consequences. Application of the criteria for evaluation of the athlete’s ECG in various populations requires special considerations based on age, ethnicity and level of sporting activity. The knowledge of the ECG findings in special populations of athletes such as the one studied contributes to the body of knowledge that may serve as substrate for future recommendations in the assessment of athlete’s ECGs. The need for inclusion of non-athletic populations in cardiovascular screening is further strengthened by the findings of this study.

### Study limitations

Several limitations of our study deserve mention. The absence of second line investigation in participants with abnormal ECG findings especially those with potentially pathologic findings; making it difficult to interpret the real diagnostic power of electrocardiography and also address the issue of false positive results. Also evaluation and subsequent analysis using a single ECG reading may have reduced the likelihood of identifying certain dynamic ECG patterns such as Brugada and Wolff-Parkinson-White syndromes. Athletes in this study had a limited duration of regular training which may have affected the development of cardiac adaptations in them and as a corollary, some controls may have been involved in some unintentional intense physical activity such as walking and/or performance activities at home. Our results therefore represent findings in African adolescent athletes in school with lower levels of regular exercise than elite athletes. The findings in this study may not be applicable to older and more professional athletes as well as those of different ethnicities.

## Conclusion

The feasibility of mass ECG screening within the school setting has been demonstrated in this study. The utility of population-based ECG screening programmes in prevention of sports-related SCD is strengthened by defined ECG criteria for differentiating physiological conditioning from cardiac pathology, taking into account variations by gender, ethnicity and various types and intensity of sporting activity. The improved accuracy is expedient to minimize errors in differentiating between physiological and pathological ECG abnormalities as this may have serious consequences such as expensive diagnostic work-up or unnecessary disqualification from competition for changes that fall within the normal range for athletes leading to significant financial and psychological loss. It is even more important to recognize signs of potentially lethal cardiovascular disorders which may erroneously be misinterpreted as normal variants of an athlete’s ECG. The weight of scientific evidence including the current study findings still suggests that a screening program inclusive of an ECG merits promotion. From this experience, it is recommended that ECG screening in addition to history and physical examination should be mandatory for all adolescent athletes as part of pre-participation evaluation before taking part in competitive sporting activities. Athletes with abnormal ECG patterns which are not considered training- related should undergo further cardiovascular evaluation before being allowed to compete. In addition, cardiovascular examination and ECG should be considered for inclusion in clinical assessment prior to school enrolment for all children, as this may be the only opportunity to detect some life threatening cardiovascular pathologies.

## Supplementary Information

**Additional file 1.**

## Data Availability

The datasets used and/or analysed during the current study available from the corresponding author on reasonable request.

## Abbreviations

AV: Atrio-ventricular
BMI: Body mass index
ECG: Electrocardiography
IRBBB: Incomplete right bundle branch block
LVH: Left ventricular hypertrophy
RBBB: Right bundle branch block
SCD: Sudden cardiac death
WHO: World Health Organization
