# Supplementary material for: Electrocardiographic pattern of apparently healthy African adolescent athletes in Nigeria
Source: BMC Pediatr. 2021 Feb 25;21:97. doi: 10.1186/s12887-021-02557-8 (PMC7905616; doi:10.1186/s12887-021-02557-8)
Supplement: Supplementary file 1 — Additional file 1. [file 12887_2021_2557_MOESM1_ESM.docx]

**STUDY PROFORMA**

**BIODATA**

1. Code number: .....................................
2. School: ................................................
3. Class: ..................................................
4. Age: ....................................................
5. Sex: a) Male b) Female
6. Ethnic group: .......................................

**HISTORY**

1. Sporting discipline: ...................................
2. Participation in competitive sport: a) Inter school b) inter house
3. Duration of regular training: No of days per week………

No of hours per week…………..

1. History of palpitation following physical activity a) yes b) no
2. History of fainting following physical activity a) yes b) no
3. History of chest pain related to physical activity a) yes b) no
4. History of breathing difficulty or fatigue related to exertion a) yes b) no
5. History of hypertension a) yes b) no
6. Previous diagnosis or treatment for heart disease a) yes b) no
7. Family history of hypertension a) yes b) no
8. Family history of heart disease a) yes b) no
9. Family history of sudden or unexplained deaths a) yes b) no
10. History of regular consumption of alcohol or tobacco a) yes b) no
11. History suggestive of chronic diseases such as kidney

Failure, liver disease, lung disease, sickle cell disease a) yes b) no

**PHYSICAL EXAMINATION**

1. Height: ..................................................
2. Weight: ..................................................
3. Body mass index: ..................................
4. Pulses: ………………………………….
5. Blood pressure: ………………………..
6. Heart murmur: ………………………...
7. Mean oxygen saturation by pulse oximetry……………………………

**ELECTROCARDIOGRAPHY DATA**

1. Heat rate: ……………………….
2. Rhythm: ……………………….
3. P axis: ……………………….
4. QRS axis: ……………………….
5. T axis: ……………………….
6. QRS T angle: ……………………….
7. PR interval: ……………………….
8. QT interval: ……………………….
9. QT_C_ interval: ……………………….
10. T wave amplitude V_5:_ …………………….
11. T wave amplitude V_6_: …………………….
12. P wave amplitude: ……………………..
13. P wave duration: ………………………..
14. Q waves: ………………………………..
15. R/S ratio in V_1:_ ………………………….
16. R/S ratio in V_2:_ ……………………….....
17. R/S ratio in V_5:_ ………………………….
18. R/S ratio in V_6:_ ………………………….
19. ST segment: …………………………….
20. T wave morphology: ………………………
21. Other abnormalities: ……………………….

ECG diagnosis: ……………………………………………………………….
